# Inhibition of Aldose Reductase Prevents Experimental Allergic Airway Inflammation in Mice

**DOI:** 10.1371/journal.pone.0006535

**Published:** 2009-08-06

**Authors:** Umesh C. S. Yadav, Kota V. Ramana, Leopoldo Aguilera-Aguirre, Istvan Boldogh, Hamid A. Boulares, Satish K. Srivastava

**Affiliations:** 1 Department of Biochemistry and Molecular Biology; 2 Department of Microbiology and Immunology, University of Texas Medical Branch, Galveston, Texas, United States of America; 3 Department of Pharmacology and Experimental Therapeutics, Louisiana State University Health Sciences Center, New Orleans, Louisiana United States of America; LMU University of Munich, Germany

## Abstract

**Background:**

The bronchial asthma, a clinical complication of persistent inflammation of the airway and subsequent airway hyper-responsiveness, is a leading cause of morbidity and mortality in critically ill patients. Several studies have shown that oxidative stress plays a key role in initiation as well as amplification of inflammation in airways. However, still there are no good anti-oxidant strategies available for therapeutic intervention in asthma pathogenesis. Most recent studies suggest that polyol pathway enzyme, aldose reductase (AR), contributes to the pathogenesis of oxidative stress–induced inflammation by affecting the NF-κB-dependent expression of cytokines and chemokines and therefore inhibitors of AR could be anti-inflammatory. Since inhibitors of AR have already gone through phase-III clinical studies for diabetic complications and found to be safe, our hypothesis is that AR inhibitors could be novel therapeutic drugs for the prevention and treatment of asthma. Hence, we investigated the efficacy of AR inhibition in the prevention of allergic responses to a common natural airborne allergen, ragweed pollen that leads to airway inflammation and hyper-responsiveness in a murine model of asthma.

**Methods and Findings:**

Primary Human Small Airway Epithelial Cells (SAEC) were used to investigate the in vitro effects of AR inhibition on ragweed pollen extract (RWE)-induced cytotoxic and inflammatory signals. Our results indicate that inhibition of AR prevents RWE -induced apoptotic cell death as measured by annexin-v staining, increase in the activation of NF-κB and expression of inflammatory markers such as inducible nitric oxide synthase (iNOS), cycloxygenase (COX)-2, Prostaglandin (PG) E_2_, IL-6 and IL-8. Further, BALB/c mice were sensitized with endotoxin-free RWE in the absence and presence of AR inhibitor and followed by evaluation of perivascular and peribronchial inflammation, mucin production, eosinophils infiltration and airway hyperresponsiveness. Our results indicate that inhibition of AR prevents airway inflammation and production of inflammatory cytokines, accumulation of eosinophils in airways and sub-epithelial regions, mucin production in the bronchoalveolar lavage fluid and airway hyperresponsiveness in mice.

**Conclusions:**

These results suggest that airway inflammation due to allergic response to RWE, which subsequently activates oxidative stress-induced expression of inflammatory cytokines via NF-κB-dependent mechanism, could be prevented by AR inhibitors. Therefore, inhibition of AR could have clinical implications, especially for the treatment of airway inflammation, a major cause of asthma pathogenesis.

## Introduction

There has been a significantly increased prevalence of asthma over the last few decades, specifically in developing countries [1]. This appears to be related to changes in the environment that affects susceptible individuals, both in the induction and worsening of established disease [2]. Epidemiological studies identified multiple interacting risk factors, including inhaled pollutants such as environmental tobacco smoke, particulate matter, oxides of nitrogen, ozone, and repeated respiratory virus exposures, which induce and/or augment reactive oxygen species (ROS) generation in the airways [3]. Although lung has excellent antioxidative system, in the presence of excessive ROS the cells become oxidatively stressed leading to loss of intracellular redox homeostasis, additional ROS production, alterations in cellular signaling and pathological processes [4], [5]. In addition, during inflammatory processes more ROS are generated by activated mast cells, macrophages, eosinophils, and neutrophils that have the potential to injure airway lining cells [6], [7]. Cellular oxidative stress plays a fundamental role in inflammation through the activation of stress kinases such as MAPKs, which comprise a large family of protein kinases including ERK1 (p44^MAPK^)/ERK2 (p42^MAPK^) and JNK, which activate redox-sensitive transcription factors such as NF-κB and AP-1 [8]. The transcription factors bind to DNA and transcribe inflammatory proteins such as cytokines, chemokines, iNOS and COX-2.

Our recent studies have shown that ROS-induced NF-κB activation is mediated by aldose reductase-catalyzed products of lipid aldehyde-glutathione conjugates [9], [10]. Aldose reductase (AR; AKR1B1), a member of aldo-keto reductase superfamily, besides reducing glucose to sorbitol, efficiently reduces lipid aldehydes and their gluthathione conjugates [11]. Most importantly, we have shown that AR-catalyzed reduced product of lipid aldehyde-glutathione conjugates such as glutathionyl-1,4-dihydroxynonane (GS-DHN) mediates NF-κB activation indicating that the inhibition of this enzyme could prevent inflammatory responses [10]. Pharmacological inhibition or siRNA ablation of AR attenuates TNF-α- and growth factor-induced IκBα phosphorylation and degradation and resultant activation of NF-κB thereby preventing the cytotoxic effects in vascular smooth muscle cells (VSMC), vascular endothelial cells (VEC) and human lens epithelial cells (HLEC) [12]–[15]. Further, our studies have shown that hyperglycemia and endotoxin-induced increase in inflammatory cytokines and chemokines in both cellular and animal models (such as restenosis, colon cancer and uveitis) is efficiently prevented by AR inhibitors [9], [16], [17]. These results suggest that AR inhibitors, initially developed as anti-diabetic drugs, could be used as therapeutic intervention to prevent inflammation [11]. AR inhibitors such as zopolrestat and fidarestat have been found to be safe and passed in FDA's Phase-I clinical trials for diabetic neuropathy but failed in Phase-III clinical trials as they have been shown to be not as effective, though they did not have any major side effects [18]. Our recent results demonstrate that AR inhibitors could have therapeutic use for the prevention and treatment of inflammatory disorders other than diabetic complications such as asthma, an airway inflammatory disease [11]. For such use a careful examination of the effect of AR inhibition in clinically relevant animal models is mandatory. We have used short ragweed (*Ambrosia artemisiifolia*) pollen extract, one of the most abundant aeroallergen that causes severe seasonal allergic symptoms in the United States, Canada and Europe, to elicit allergic responses via induction of oxidative stress that mimics airway inflammation in humans [19]–[21]. In the present study, we investigated whether treatment with AR inhibitors could prevent alterations in the cytokine and chemokine levels in a cellular model using human SAEC and a clinically relevant mice model which mimics allergic airway inflammatory conditions in humans. Our results indicate that AR inhibitors are powerful repressors of the expression of major cytokines and chemokines in a mouse model of ragweed–induced allergic airway inflammation that leads to asthma.

## Results

### AR Inhibition prevents RWE-Induced apoptosis in SAEC

**Figure 1:**
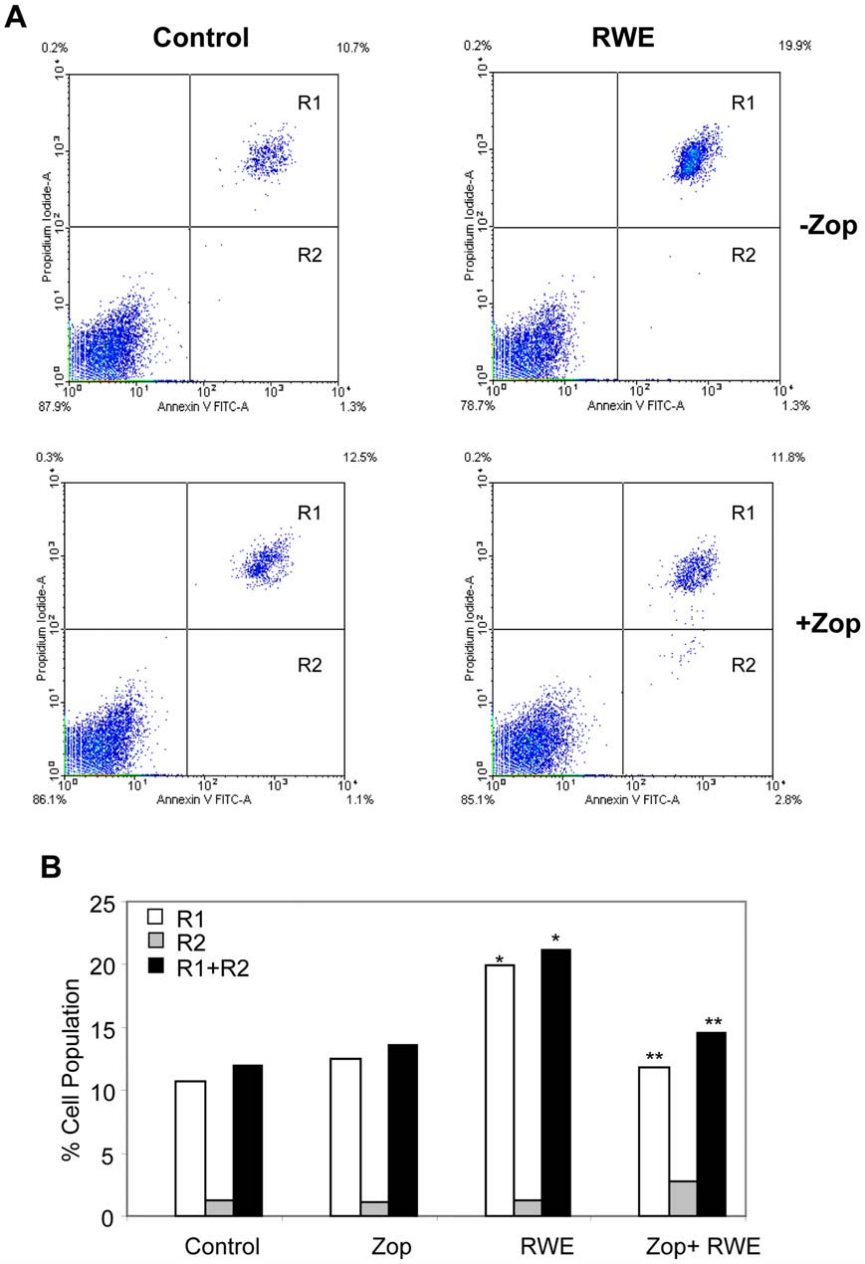
Inhibition of AR prevents RWE-induced apoptosis and cell death in SAEC. (A) Growth-arrested SAEC, pretreated without or with zopolrestat (20 µM), were incubated with 150 µg/ml of RWE for 18 h to induce apoptotic cell death. The cells were stained with annexin-V FITC (FL-1) and propidium iodide (PI) (FL-2). R1 denotes dead cells (PI positive), R2 represents early apoptotic cells (annexin-V positive) and R1+R2 represents total dead cells. (B) The data from (A) has been plotted as bar diagram (n = 4, ^*^ *p*<0.01 Vs Control; ^**^ *p*<0.01 Vs RWE). RWE, ragweed pollen extract; zop, zopolrestat.

The airway epithelium plays an important function as a barrier to foreign particles, pollens, spores of fungi and other xenobiotics which disturb cellular redox homeostasis leading to apoptosis of airway epithelial cells [22]–[24], [29], [33]. Hence, we first determined the effect of AR inhibition on RWE–induced cell death of SAEC by Annexin-V binding assay. Annexin-V binds to the inverted phosphotedylserine in the cells undergoing apoptosis. Propidium-iodide (PI) was used as the indicator of the cell mortality. In SAEC, RWE-treatment caused increased cell death (over 50%) in 18 h as compared to the control cells. Pre-incubation of the cells with AR inhibitor, zopolrestat, significantly (p<0.01) prevented RWE-induced cell death by >80% (Fig. 1B). Under similar conditions, AR inhibition alone did not cause apoptosis of SAEC.

### AR inhibition decreases RWE-induced ROS formation in SAEC

**Figure 2:**
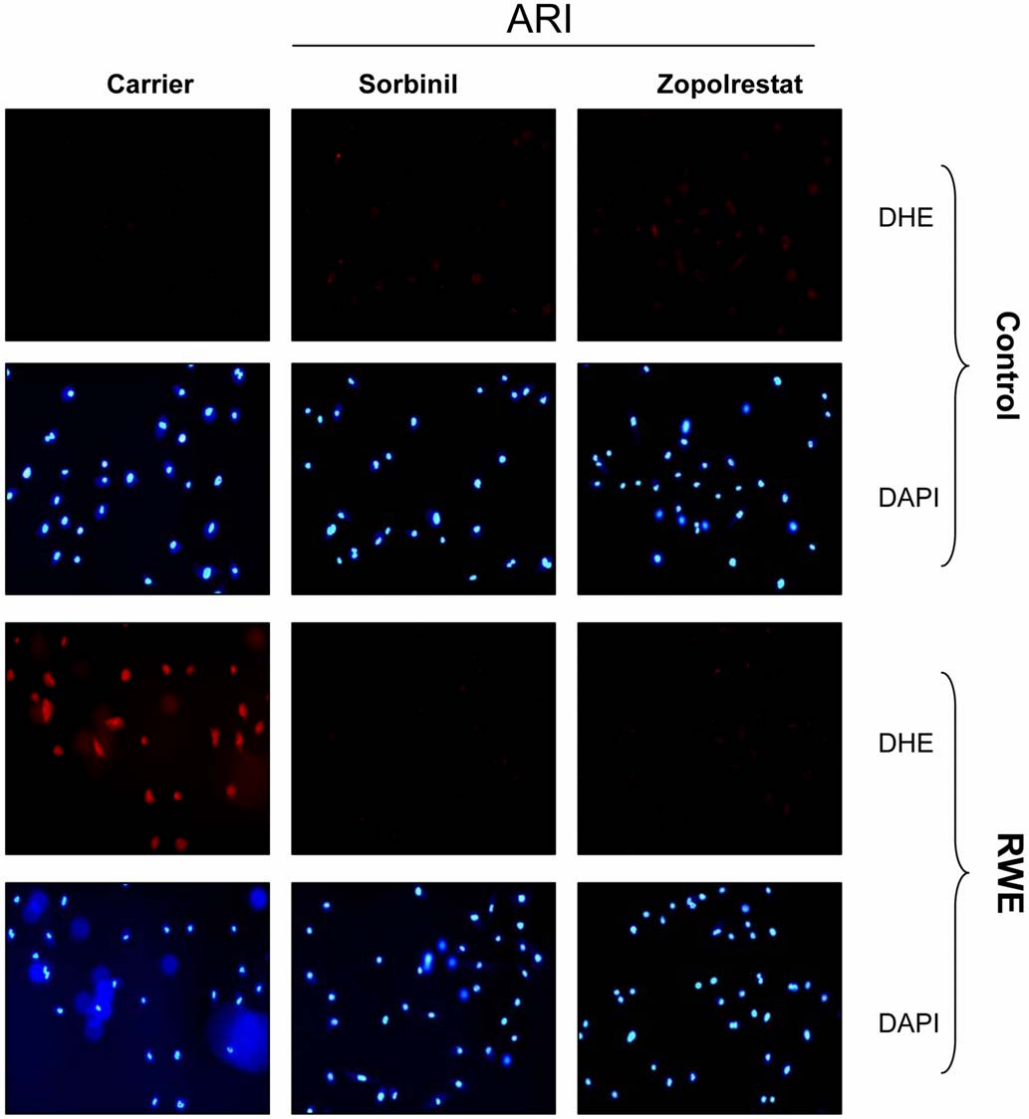
Inhibition of AR prevents RWE-induced ROS generation in SAEC. Approximately 1×10^5^ cells were seeded on 2-chambered slides and starved in serum-free basal medium with or without AR inhibitors, sorbinil or zopolrestat, for 24 h. The cells were treated with RWE (150 µg/ml) for 16 h. The SAEC were washed with cold PBS (pH 7.2) and stained with ROS-sensitive dye, dihydroethidium (DHE) for 15 min at 37°C. The cells were washed again and mounted with floursave (with diamidino-2-phenylindole (DAPI)) mounting medium. Photomicrographs were acquired by a fluorescence microscope (Nikon). A representative picture is given (n = 4); Magnification 200×. ARI, aldose reducatse inhibitor.

It has been reported that addition of RWE to cultured epithelial cells or airways increases oxidative stress levels within minutes after exposure [21], [25]. To examine the nature of the RWE-induced decrease in SAEC viability, we measured the level of ROS in RWE-induced SAEC and whether AR inhibitors could prevent it. As shown in Fig. 2, RWE (150 µg/mL) caused increase in cellular ROS levels as evident by increased fluorescence by ROS sensitive dihydroethidium (DHE). Pre-incubation of SAEC with two different AR inhibitors, sorbinil or zopolrestat, prevented these changes (Fig. 2). Under the similar conditions, AR inhibition alone caused no significant change in the ROS levels in SAEC. These results suggest that inhibition of AR increased antioxidant potential of cells and prevented RWE-induced increase in ROS and resultant cell death.

### AR inhibition prevents RWE-induced production of inflammatory cytokines and chemokines in SAEC

**Figure 3:**
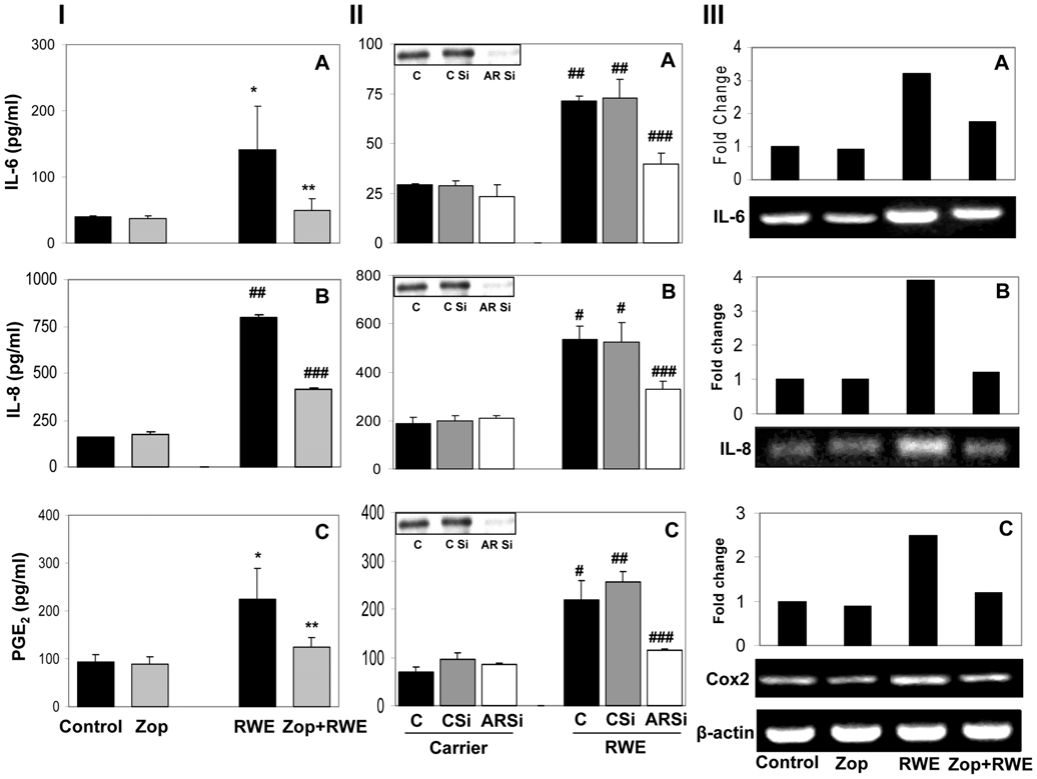
Pharmacological inhibition or genetic ablation of AR prevents RWE-induced secretion and expression of inflammatory markers in SAEC. Approximately 2×10^5^ SAEC were seeded in 6-well plates and incubated until 80% confluency. (I). The cells then starved in serum-free basal medium with or without AR inhibitor, zopolrestat, at 37°C for 24 h. The cells were incubated with RWE (150 µg/ml) for an additional 24 h. The medium was harvested, centrifuged and supernatant was used for the determination of IL-6 (A), IL-8 (B) and PGE_2_ (C) with respective ELISA kits following supplier's manuals. Bars represent Mean±SD (n = 4); ^*^ *p*<0.05 Vs Control; ^##^ *p*<0.001 Vs Control; ^###^ *p*<0.01 Vs RWE alone ^**^ *p*<0.05 Vs RWE alone. zop, zopolrestat; RWE, ragweed pollen extract. (II). SAEC were transfected with AR-SiRNA or scrambled-SiRNA in basal medium. After 48 h, medium was replaced with fresh basal medium and the cells were treated with RWE (150 µg/ml) and incubated for an additional 24 h. The medium was harvested, centrifuged and supernatant was used for the determination of IL-6 (A), IL-8 (B) and PGE_2_ (C) with respective ELISA kits following supplier's manuals. Bars represent Mean±SD (n = 4); ^#^ *p*<0.01 Vs Control; ^##^ *p*<0.001 Vs Control; ^###^ *p*<0.01 Vs RWE alone. C, control; CSi, scrambled siRNA; ARSi, aldose reductase siRNA; zop, zopolrestat; RWE, ragweed pollen extract. (III). SAEC were starved in serum-free basal medium with or without zopolrestat for 24 h. The cells were treated with RWE (150 µg/ml) for 6 h. Total RNA was extracted as described in the methods and IL-6 (A), IL-8 (B) and COX-2 (C) mRNA expression was determined using Qiagen RT-PCR kit and β-Actin was used as loading control. A representative gel showing amplified PCR products is shown (n = 3). zop, zopolrestat; RWE, ragweed pollen extract.

Since RWE is known to elevate the levels of inflammatory markers in the airway epithelial cells that cause inflammation and aggravate the allergic condition [25], we next examined the effect of AR inhibition on the RWE-induced increase in the levels of selected inflammatory markers in SAEC culture medium. As shown in Fig. 3(I) A and B, treatment of SAEC with RWE (150 µg/mL) for 24 h caused 3 and 4-fold increase in the synthesis of IL-6, and IL-8, respectively, and inhibition of AR by pharmacological agents significantly prevented the increase. A more than 2-fold increase in the RWE-induced PGE_2_ levels in SAEC was also significantly (>75%) prevented by AR inhibition (Fig. 3(I) C).

Although zopolrestat is a specific inhibitor of AR, to rule out its non-specific response in the biological system, we ablated AR message in SAEC by antisense oligonucleotides (AR siRNA) and studied whether phenotypic absence of AR will have similar effects in SAEC as did AR inhibitor in protection against RWE-induced inflammation. Transient transfection of SAEC with AR siRNA abolished AR protein by >95% (Fig. 3(II) A–C, inset) while with scrambled siRNA oligonucleotides AR expression did not change. We observed that siRNA ablation of AR also significantly prevented RWE-induced synthesis of cytokines such as IL-6, and chemokines IL-8 and PGE_2_ in SAEC (Fig. 3(II) A–C). We further examined the effects of AR inhibition on the expression of inflammatory markers at RNA levels using quantitative RT-PCR. As shown in Fig. 3(III) A&B, treatment of SAEC with RWE caused a 3–4 fold increase in the expression of IL-6 and IL-8 mRNA level and zopolrestat prevented it by >70% suggesting that AR could regulate the transcriptional activation of inflammatory marker genes. Also, since PGE_2_ is synthesized by inducible cycloxygenase (COX)-2, we determined the effect of AR inhibition on the transcriptional activation of COX-2 by quantification of its mRNA in response to RWE in SAEC by RT-PCR. As shown in Fig. 3(III) C, RWE significantly increased the mRNA levels of COX-2 in SAEC and zopolrestat prevented it by more than 60%. These results suggest that AR regulates the synthesis as well as expression of inflammatory markers.

### AR inhibition prevents RWE-induced expression of inflammatory markers in SAEC

**Figure 4:**
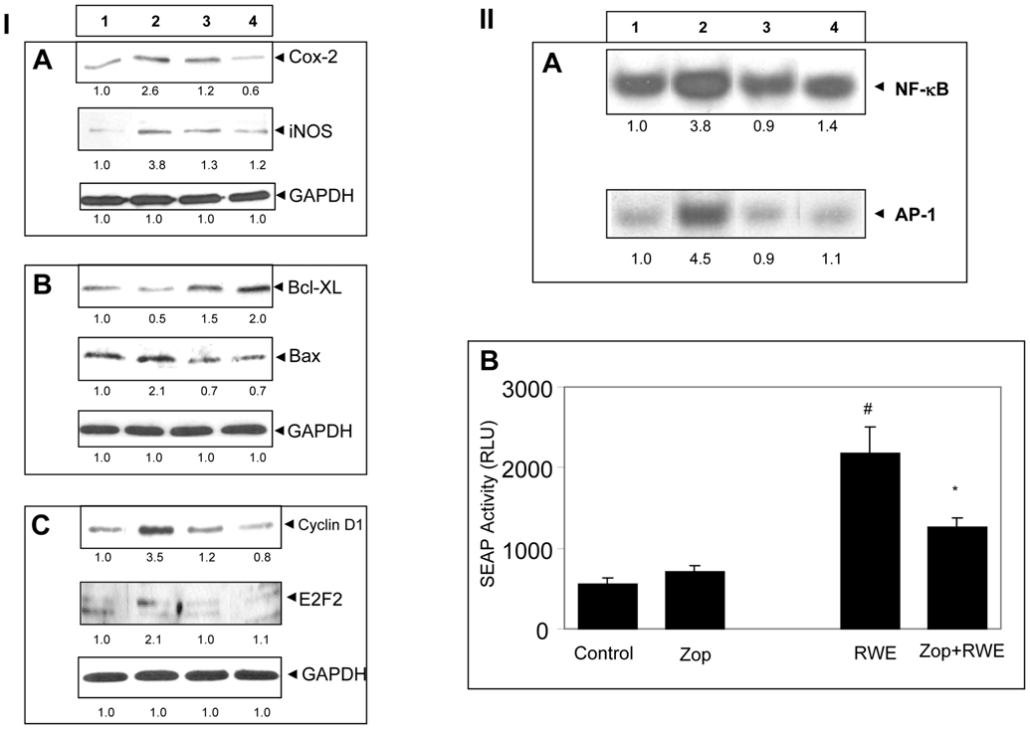
Inhibition of AR prevents RWE-induced expression and activation of inflammatory, apoptotic and cell cycle proteins and redox-sensitive transcription factors NF-κB and AP-1 in SAEC. (I) Approximately 2×10^5^ SAEC were seeded in 6-well plates and incubated until 80% confluency. The cells were starved in serum-free basal medium with or without zopolrestat for 24 h. The cells were treated with RWE (150 µg/ml) for 24 h and cell lysate was prepared. Immunoblotting was performed using antibodies against (A) COX-2, iNOS, (B) Bcl-XL, Bax, and (C) Cyclin D1, E2F2 to determine the expression of various proteins. GAPDH was used as loading control. Representative blots are shown (n = 3), numbers below the blots represent fold changes. Lanes: 1, control; 2, RWE; 3, control+zop; 4, RWE+zop. zop, zopolrestat; RWE, ragweed pollen extract. (II) (A) For EMSA, approximately 2×10^6^ SAEC were seeded in T-150 cm^2^ flasks and incubated until 80% confluency. The cells were starved in serum-free basal medium with or without zopolrestat for 24 h. The cells were treated with RWE (50 µg/ml) for 3 h. Nuclear extract was prepared and EMSA was performed to assess the DNA binding activity of NF-κB and AP-1. Lanes: 1, control; 2, RWE; 3, control+zop; 4, RWE+zop. zop, zopolrestat; RWE, ragweed pollen extract. (B) Approximately, 1×10^5^ SAEC were plated in 24-well plate and growth-arrested by preincubatin in serum-free basal medium with AR inhibitor or carrier for 24 h followed by transfection with NF-κB-pSEAP vector or control (pTAL) vector. After 6 h, transfected cells were incubated with RWE (50 µg/ml) for 48 h. Medium was collected, cleared by centrifugation and NF-κB-dependent reporter SEAP activity was measured by chemiluminescence's method essentially as described by the manufacturer. Bars represent Mean±SD (n = 4). ^#^ *p*<0.001 Vs. Control; ^*^ *p*<0.01 Vs RWE. zop, zopolrestat; RWE, ragweed pollen extract.

Since biosynthesis of PGE_2_ and NO from their precursors is catalyzed by COX-2 and iNOS enzymes respectively, we next examined the effect of AR inhibition on RWE-induced COX-2 and iNOS expression in SAEC by immunoblotting. As shown in Fig. 4(I) A, treatment of SAEC with RWE significantly (∼3-folds) increased COX-2 and iNOS protein expression and pre-treatment of SAEC with AR inhibitor, zopolrestat, significantly (>90%) prevented the increase. This indicates that RWE-induced COX-2 and iNOS overexpression is mediated by AR, which is obligatory for RWE-induced PGE_2_ and NO production that exacerbate allergic inflammation.

### AR inhibition prevents RWE-induced alterations in pro- and anti-apoptotic proteins in SAEC

Since apoptosis is regulated by the fine balance between the pro-apoptotic and anti-apoptotic proteins, we next examined the effect of AR inhibition on the expression of these proteins. As shown in Fig. 4(I) B RWE caused more than 2-fold increased expression of pro-apoptotic protein Bax while the expression of anti-apoptotic protein Bcl-XL decreased by 50%. The over-all ratio of pro- and anti-apoptotic proteins in control cells was 1 which increased significantly (to approximately 4) in RWE-treated cells. Inhibition of AR not only controlled the expression of these proteins but it also maintained the ratio to approximately one. These results suggest that AR inhibition prevented the RWE-induced alteration in the ratio of pro-and anti-apoptotic proteins and thereby promoted cell survival and inhibited apoptosis in these cells.

### AR inhibition prevents RW-induced over-expression of cell cycle proteins in SAEC

As we showed above RWE induces epithelial cell injury and apoptosis that affects the cell cycle progression. The number of cells entering the cell division cycle is regulated by a fine balance of cell cycle proteins. We therefore examined whether AR inhibition will affect the level of cell cycle proteins in RWE-treated SAEC. As shown in Fig. 4(I) C, RWE caused >3.5 fold increased expression of cyclin D1 and >2.5 fold increased expression in E2F2 proteins. Inhibition of AR by zopolrestat significantly (>90%) prevented the increase in the expression of cell cycle proteins. These results suggest that inhibition of AR is critical to maintaining the cell cycle under oxidative stress and prevents cells from undergoing apoptosis.

### AR inhibition prevents RWE-induced activation of NF-κB and AP1

We next examined the effect of AR inhibition on RWE-induced activation of NF-κB and AP1 because these transcription factors are primarily responsible for the transcription of various inflammatory markers. As shown in Fig. 4(II) A, RWE caused approximately 4-fold activation of NF-κB and AP-1 and zopolrestat significantly (>80%) prevented RWE-induced NF-κB activation and nuclear translocation. Zopolrestat alone did not affect the basal NF-κB activity in the SAEC. To further confirm NF-κB activation by RWE, we used NF-κB-dependent secretory alkaline phosphatase (SEAP) reporter assay and found that RWE significantly (>3-fold) induced NF-κB-dependent SEAP activation in SAEC and AR inhibitor, zopolrestat, caused >60% inhibition (Fig. 4(II) B). However, zopolrestat alone did not affect the NF-κB-SEAP activity. These results validated our measurement of DNA binding activity of NF-κB by gel-shift assay. Based on these observations, we conclude that inhibition of AR prevented RWE-induced activation of NF-κB, which could activate the expression and synthesis of inflammatory markers in SAEC.

### AR inhibition prevents RWE-induced accumulation of eosinophils in mice airway

**Figure 5:**
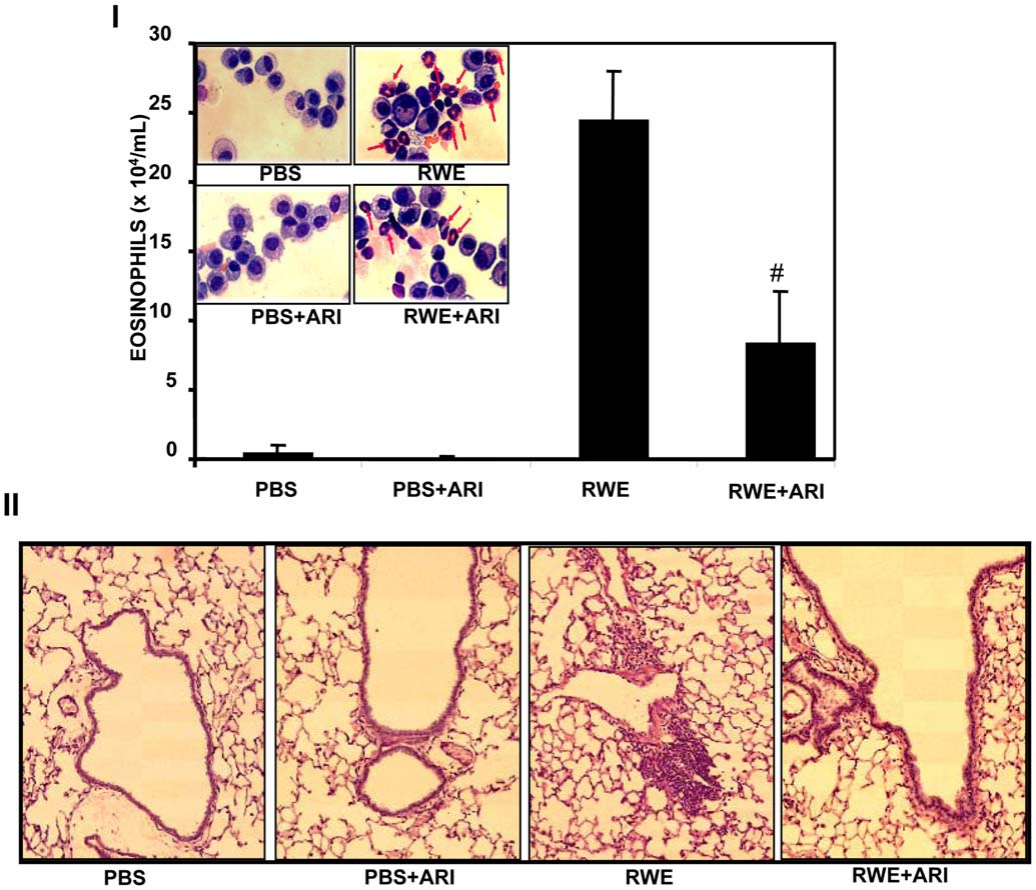
AR inhibition prevents RWE-induced accumulation of eosinophils in mice. (I) Determination of eosinophils in BAL. The Balb/c mice were sensitized and challenged with PBS, RWE, ARI alone, or ARI+RWE and 72 h later lungs were lavaged with ice-cold PBS and differential cell counts were performed on cytocentrifuge preparations stained with hematoxylin and eosin. Bars represent Mean±SD (n = 6–8), ^#^ *p*<0.002 Vs RWE. Inset: representative fields of hematoxylin and eosin preparations were photographed (magnification ×90). (II) Determination of eosinophils in peribronchial and subepithelial regions of lung. Sensitized BALB/c mice were challenged as in (I), 72 h later lungs were excised, fixed with 4% paraformaldehyde, embedded in paraffin, and sectioned to 5 µm. Sections were stained with hematoxylin and eosin. Photomicrographs were acquired by photometrix CoolSNAP Fx camera mounted on a NIKON Eclipse TE 200 UV microscope. A representative field for each group is shown (magnification: ×30). PBS, phosphate buffer saline; RWE, ragweed pollen extract; ARI, aldose reductase inhibitor

Since inflammatory response of RWE challenge to SAEC was blocked significantly by AR inhibition, we tested whether this approach would work in the animal model as well. We therefore sensitized and challenged the BALB/c mice with RWE- or carrier-treated without or with AR inhibitor. As shown in Fig. 5(I) there was a robust airway inflammation as measured by accumulation of inflammatory cells in bronchoalveolar lavage (BAL) fluid and sub-epithelium in RWE-sensitized and - challenged animals. In the mice treated with AR inhibitor there was significantly (p<0.002) less inflammation as determined by the number of eosinophils in BAL fluid. Similarly, perivascular and peribronchial inflammation and cell composition in the BAL fluid induced by RWE challenge was significantly prevented by AR inhibitor treatment (Fig. 5(II)).

### AR inhibition prevents RWE-induced mucin levels and hyperreactivity in mice airway

**Figure 6:**
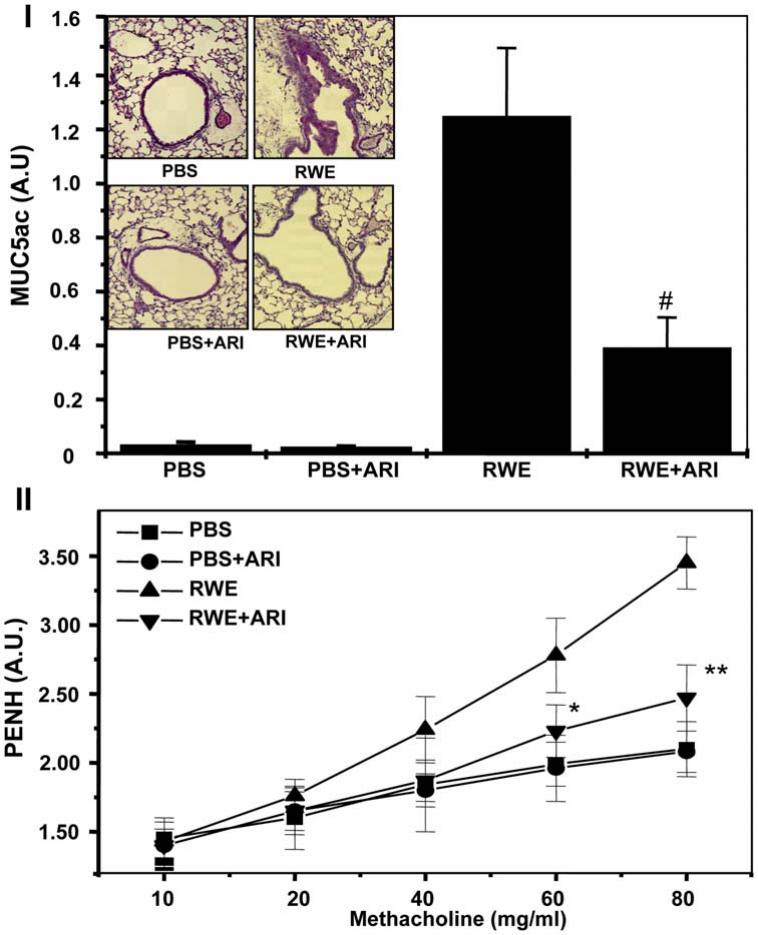
AR inhibition prevents RWE-induced mucin production and methacholine –induced airway hyper-responsiveness in mice. (I) The lungs were lavaged with ice-cold PBS 72 h after RWE challenge. Mucin levels in the BAL were determined by ELISA using anti-MUC5ac monoclonal antibody. Bars represent Mean±SD (n = 6), ^#^ *p*<0.003 Vs RWE. Inset: sections were stained with periodic acid Schiff (PAS)-stain to visualize mucin producing cells. A representative field is shown (magnification: ×30). (II) The changes in pause of breathing ‘enhanced pause’ (PENH) as an index of airway obstruction were measured by whole-body plethysmography. Mice were placed in a barometric plethysmographic chamber and Penh was determined and plotted against the increasing concentration of methacholine. Bronchopulmonary resistance was expressed as enhanced pause = (expiratory time/relaxation time)-1)×(peak expiratory flow/peak inspiratory flow). Each data point represents mean±SD of 6 to 8 mice for each group. ^*^p<0.05, ^**^p<0.01 Vs RWE. ARI, aldose reductase inhibitor; RWE, ragweed pollen extract; PBS, phosphate buffer saline; PENH, enhanced pause.

Excessive mucin production by airway epithelium is characteristic of allergic asthma and its prevention is the main goal to treat allergic episodes in susceptible individuals [26]. Therefore, we next examined the mucin levels in the BAL fluid by ELISA using anti-MUC5ac monoclonal antibodies and found that mucin levels increased ∼25-folds in RWE-challenged mice as compared to control and AR inhibition prevented it significantly (p<0.003). Similarly, mucin production in the epithelial cells as assessed by periodic acid Schiff (PAS)-staining of lung sections (Fig. 6(I), inset) was also prevented by AR inhibition. In addition, whole body unrestrained plethysmography was used to quantitatively measure airway responsiveness in mice after methacholine challenge. As shown in Fig. 6(II), enhanced pause (Penh) elevated dose-dependently in response to methacholine challenge as compared to control mice treated with PBS alone. Treatment of mice with AR inhibitor decreased the Penh values significantly (p<0.01) from methacholine (80 mg/ml) alone-challenged mice. These results indicate that AR inhibition significantly prevented the patho-physiological effects of allergic asthma in murine model.

### AR inhibition prevents RWE-induced cytokines and chemokines secretion in mice airway

Th2 type cytokines such as IL-4, IL-5 and chemokines have been implicated in the pathology of allergic asthma [27]. Also, the expression of Th2 cytokine mRNA have been shown to increase during allergic inflammation [28]. Therefore, we determined the effect of AR inhibition on the RWE-induced expression of Th2 type cytokines and chemokines in lung by quantitative RT-PCR. As shown in Table 1, RWE challenge caused increased expression of interleukin (IL)-4, IL-5, IL-6, IL-10 and IL-13, and chemokines such as Chemokine (C-C motif) ligand (CCL)-4 and CCL-5 which was significantly prevented by AR inhibition. There was no change in the level of expression of these cytokines and chemokines in AR inhibitor alone treated mice lungs.

**Table 1:** AR inhibition prevents RWE-induced expression of cytokine and chemokines in mice lungs.

Cytokine and chemokines	Abbrev.	ARI alone (n = 5)	RWE alone (n = 5–6)	RWE+ARI (n = 7)	P value
Interleukin 4	IL-4	1.07±0.13	15.37±0.71	3.84±0.44	*P* = 0.0012
Interleukin 5	IL-5	1.09±0.09	6.84±0.63	2.84±0.14	*P* = 0.0023
Interleukin 6	IL-6	0.87±0.16	3.11±0.21	1.94±0.53	*P* = 0.017
Interleukin 8	IL-8	0.97±0.03	4.58±0.77	2.18±0.31	*P* = 0.0019
Interleukin 10	IL-10	0.93±0.011	6.64±0.21	3.18±0.21	*P* = 0.022
Interleukin 13	IL-13	1.17±0.23	9.14±0.29	3.18±0.66	*P* = 0.017
Interleukin 17	IL-17	1.09±0.019	6.24±0.27	2.50±0.10	*P* = 0.031
Interleukin 18	IL-18	1.17±0.011	2.64±0.47	1.64±0.47	*P* = 0.027
Chemokine (C-C motif) ligand 4	CCL4	1.01±0.09	5.18±0.19	1.18±0.59	*P* = 0.019
Chemokine (C-C motif) ligand 5	CCL5	1.12±0.22	7.38±0.29	4.18±0.79	*P* = 0.033
Tumor necrosis factor alpha	TNF-α	0.96±0.14	4.83±0.53	2.33±0.41	*P* = 0.029

Total RNA was isolated from homogenized lungs (n = 5–7), one microgram of total RNA from each sample was transcribed into first-strand cDNA and quantitative RT-PCR was conducted for selected genes using specific forward and reverse primers. The levels of RNA for the target sequences were determined by melting curve analysis. The values presented here are fold-change over the control. RWE, ragweed pollen extract; ARI, aldose reductase inhibitor.

## Discussion

Among many factors, weed pollens are associated with the allergic asthma causing loss of human work hours and severe health problems, sometimes leading to life threatening conditions. Ragweed pollens are among many seasonal weed pollens that have been shown to induce severe allergic response in susceptible individuals. Ragweed pollens or their extract have been shown to possess intrinsic ROS producing machinery due to their intrinsic NAD(P)H oxidase [25], [29]. We have shown that RWE administration enhances ROS levels in the airway epithelium of murine lungs and also increases the levels of oxidized glutathione (GSSG), 4-HNE, and malondialdehyde in the lung tissue [25]. Also, ROS induced by environmental pollutants including diesel exhaust and ozone have been recognized as the mediator of inflammation in the airways causing and exacerbating allergic asthma [30]–[33]. Thus, it is well recognized that oxidative stress-induced changes in airway epithelial cells physiology plays a central role in innate and adaptive immune responses as well as mucosal inflammation [34]. Epithelial cells produce a large number of proinflammatory cytokines and chemokines in response to allergens [27], [34] which further induce ROS - formation resulting in profound oxidative stress.

It is well established that oxidative stress in airway epithelium stimulates and initiates a molecular signal cascade leading to the activation of NF-κB [35], [36] which translocates to the nucleus. In the nucleus NF-κB binds to DNA and transcribes inflammatory markers such as interleukin (IL)-6, IL-8, macrophage inflammatory protein 2 (MIP-2), granulocyte macrophage colony-stimulating factor (GM-CSF), intercellular adhesion molecule-1 (ICAM-1), COX-2 and iNOS, which besides directly causing inflammation also promote inflammatory cells infiltration [37]–[42]. The infiltrated eosinophils, macrophages and T lymphocytes further secrete various cytokines, chemokines and other inflammatory markers that cause airway narrowing, hyperresponsiveness and remodeling [41]–[43]. Thus, although the molecular events associated with respiratory allergy and asthma are well understood, the cure for this respiratory disorder which affects approximately 20% of the US population is still elusive, especially because of the lack of understanding how ROS mediates allergic stimulant-induced activation of NF-κB. Here, we show for the first time that inhibition of a polyol pathway enzyme, AR, could attenuate RWE-induced airway inflammation and block the inflammatory cells infiltration and expression of inflammatory markers in the airway epithelial cells.

We have shown earlier that AR inhibition prevents oxidative stress-induced signaling cascade that activates transcription factors such as NF-κB and AP-1 [9]–[14] which could prevent the ROS-mediated airway inflammation. The airway epithelial cells are known to produce ROS in response to many stimuli including weed pollens and virus [25], [44]. The locally produced ROS oxidize the cell membrane phospholipids and generate lipid hydroperoxides which impair membrane function such as membrane transport, dysfunction of channel proteins, and loss of fluidity leading to tissue injury [3]. The lipid peroxidation products, including the most abundant and reactive lipid aldehydes such as 4-HNE, have been shown in the airways of experimental animals [3], [25]. 4-HNE is known to induce various cellular events including activation of signaling cascade leading to cytotoxicity [45], [46]. Also, 4-HNE readily forms conjugates with glutathione (GS), GS-HNE. In vascular smooth muscle cells and murine macrophages we have recently shown that AR-catalyzed reduced product of GS-HNE conjugates, glutathionyl-1,4-dihydroxynonane (GS-DHN), caused activation of NF-κB-dependent signaling, leading to cytotoxicity [10], [47]. These data designated a novel role for AR-catalyzed reduced product, GS-DHN, which could be responsible for activation of inflammatory signaling during oxidative stress induced by various stimuli including pathogens, allergens and environmental pollutants. These results indicate that inhibition of AR that blocks the reduction of glutathione-lipid aldehyde conjugate could be a novel approach to prevent inflammation.

Antioxidants and ROS scavengers such as ascorbic acid, N-acetyl-L-cysteine (NAC), and superoxide dismutase (SOD) have been shown to be effective in controlling ROS-mediated airway inflammation [48], [49]. Increased dietary intake of ascorbic acid has been shown to improve lung functions in asthma patients [50]. Similarly, NAC has been shown to have protective effects both *in vitro* and *in vivo* against oxidative stress in airway inflammation [51]–[53]. These evidences suggest that quenching ROS or blocking its effect on airway epithelial cells could be a potential strategy to prevent inflammation and tissue injury during allergic asthma. We observed that in SAEC, AR inhibition significantly prevents ROS formation by RWE challenge and thereby prevents ROS-induced cytotoxicity, including cellular apoptosis. These results suggest that AR inhibition could successfully attenuate the effect of ROS as well as ROS formation in SAEC.

Increased levels of inflammatory markers including cytokines such as interleukins (IL-6), chemokines such as IL-8 and other inflammatory mediators such as PGE_2_ by airway epithelial cells are the hallmarks of inflammatory response in allergic asthma [54]. Primarily, these inflammatory markers are released by airway epithelial cells in response to allergens in order to remove and dispose off the allergens by recruiting immune cells. However, excessive allergen exposure causes an overwhelming response by epithelial cells resulting in increased ROS formation leading to activation of redox-sensitive transcription factors such as NF-κB and AP-1 [36], [42] which transcribe various inflammatory markers that cause cytotoxicity and damage to airway resulting in the pathogenesis of allergic asthma. Therefore, blocking the synthesis and release of the inflammatory markers could potentially prevent asthma pathogenesis. Anti-interleukin antibody treatment has been suggested to have considerable therapeutic potential for asthma and allergy [55]–[58]. Similarly, various studies have shown increased iNOS expression in airway epithelium of asthma patients [59], [60]. COX-2 has also been found to be elevated during asthma [60]. COX-2 and iNOS catalyze oxidative stress-inducible production of PGE_2_ and NO, respectively which play important role in the pathogenesis of asthma and have been suggest as possible therapeutic targets [60]. In the present study we have shown that pharmacological inhibition as well as genetic ablation of AR message significantly (70–90%) inhibited RWE-induced expression of these inflammatory markers in SAEC which suggests that AR is a key mediator in RWE-induced inflammation and inhibition of AR, that we have shown earlier prevents the oxidative stress-induced generation of COX-2 and iNOS, would prevent allergen-induced airway inflammation.

Since our ultimate goal is to develop an effective therapeutic intervention to allergic respiratory disorders, we tested whether significant prevention of inflammatory response of RWE challenge in *in-vitro* cellular model would work in the animal model of allergic asthma as well. The recruitment of eosinophils in the airway plays an important role in the pathogenesis of allergic asthma [41], [43]. Studies with RWE–challenged mice have shown that inflammatory cells including eosinophils infiltrate the airway [25], [49]. A study using suicidal signals specific to eosinophils in mice showed that eosinophils infiltration is mandatory for the pathology of asthma [61]. We, using murine model of RWE-induced airway inflammation, observed that treatment with AR inhibitor significantly prevented the airway inflammation and recruitment of eosinophils in BAL fluid and subepithelial spaces. In addition, RWE-induced hyperresponsiveness and excessive mucin production by airway epithelium and Th2 type cytokines and chemokines gene expression in sensitized mice were also significantly prevented by AR inhibition. These data with animal model of RWE-induced airway inflammation confirm our *in-vitro* finding with human primary airway epithelial cells and suggest that AR inhibitors could be initiated for controlled clinical trial in allergic asthma patients. A recent report suggests that AR inhibitors such as zopolrestat and fidarestat have been found to be safe in clinical trials and did not show any major side effects [18].

Thus, with predicted increase in the ragweed pollen production under increased environmental CO_2_ condition in future climate there is strong likelihood of prevalence of allergic disorders [62]. In view of limitations and side effects of present day anti-inflammatory medication to treat allergic disorders, it is imperative to search for a pharmacological agent with minimal side effects. The evidences presented in this report indicate that AR inhibitors could be a novel approach to control the allergen-induced inflammation in the airway epithelial cells. In summary, we have presented the evidence that AR mediates the respiratory inflammatory response during ragweed pollen-induced allergic asthma and have provided a novel concept that inhibition of AR could be a therapeutic approach in the treatment of allergic airway inflammation.

## Materials and Methods

### Reagents

Small airway epithelial basal medium (SABM), and small airway epithelial growth media (SAGM™) bulletkit; and one Reagentpack™ containing Trypsin 0.025%/EDTA 0.01%, Trypsin neutralizing solution and HEPES buffered saline solution were purchaged from Cambrex Bio Sciences Walkersvillle, Inc. (Walkersville, MD). Sorbinil and Zopolrestat were obtained as gift from Pfizer (New York, NY). Dimethyl sulfoxide (DMSO) was obtained from Fischer scientific (Pittsburg, PA). Ragweed pollen extract (RWE) was purchased from Greer's laboratory (Lenoir, NC). Nitrite/Nitrate and PGE_2_ assay kits were obtained from Cayman Chemical Inc (Ann Arbor, MI). Human IL-6 and IL-8 ELISA kits were from Diaclone (Stamford, CT) and R&D systems, respectively. Antibodies against COX-2, iNOS, Bcl-XL, Bax, GAPDH, cyclin-D1 and E2F2 were from Santa Cruz Biotechnology Inc. (Santa Cruz, CA); and antibodies against phospho-IκB were from Cell signaling (Danvers, MA). Dihydroethidium (DHE) fluorescent dye was purchased from Molecular Probes, Invitrogen (Carlsbad, CA) and polyclonal antibodies against human recombinant AR were made for us by Alpha diagnostic intl. (San Antonio, TX). The reagents used in the electrophoretic mobility shift assay (EMSA) and Western blot analysis were obtained from Sigma. All other reagents used were of analytical grade.

### Cell Culture

Primary human Small Airway Epithelial Cells (SAEC) obtained from Cambrex Bio Science Walkersville, Inc. (Walkersville, MD) were normal human SAEC harvested from distal airspace of 18 yrs old male donor. The cells were cultured according to the supplier's instructions at 37°C in humidified atmosphere containing 95% air and 5% CO_2_ in small airway epithelial basal medium (SABM) with supplements containing 52 µg/ml bovine pituitary extract, 0.5 ng/ml human recombinant epidermal growth factor (EGF), 0.5 µg/ml epinephrine, 1 µg/ml hydrocortisone, 10 µg/ml transferrin, 5 µg/ml insulin, 0.1 ng/ml retinoic acid (RA), 6.5 ng/ml triiodothyronine, 50 µg/ml Gentamicin/Amphotericin-B (GA-1000), and 50 µg/ml fatty acid-free bovine serum albumin (BSA).

### Annexin-V Staining and Flow Cytometry

Approximately 2×10^5^ SAEC per well were plated in 6-well plates in triplicate for each group. The medium was replaced with serum-free SABM with or without zopolrestat (20 µM) and incubated for 24 h. The cells were treated by RWE (150 µg/mL) and incubated for 18 h. Apoptotic cell death was examined using the annexin-V-FITC/propidium iodide (PI) (molecular probes, Invitrogen) according to the manufacturer's instructions. Twenty thousand events were acquired for each sample and analyzed by flow cytometry using the LYSIS II software (FACScan, BD Pharmingen).

### In situ detection of superoxide

Dihydroethidium (DHE, Molecular Probes) staining was carried out to assess the ROS production. Briefly, Approximately 1×10^5^ SAEC were seeded on chambered slides and starved in serum-free SABM with or without AR inhibitor for 24 h. The cells were treated with RWE (150 µg/mL) for 16 h. SAEC were rinsed with cold PBS and incubated in PBS containing DHE (2.5 µmol/L) at 37°C for 15 minutes. Cells were rinsed in PBS and mounted with mounting medium with DAPI (Vector Laboratories Inc., Burlingame, CA). The image of ethidium staining was observed and photomicrographs were acquired with a Nikon epifluorescence microscope with a 585 nm long-pass filter. Generation of superoxide in the cells was demonstrated by strong red fluorescent labeling.

### Electrophoretic Mobility Shift Assay (EMSA)

Approximately 90% confluent SAEC in T-150 cm^2^ culture flasks were incubated with AR inhibitor or carrier for 24 h in starving medium, followed by treatment with RWE (50 µg/ml) for 2 h at 37°C. The nuclear extracts were prepared as described [63]. The Consensus oligonucleotides for NF-κB and AP-1 transcription factors were 5′-end labeled using T4 polynucleotide kinase. EMSA was performed as described earlier [64].

### NF-κB-dependent secretary alkaline phosphatase expression (SEAP) reporter Assay

Approximately 1×10^5^ SAEC per well were plated in 24-well plates, serum-starved in SABM for 24 h with or without AR inhibitor, zopolrestat (20 µM), and transiently transfected with pNF-κB-secretory alkaline phosphatase (SEAP) construct or control plasmid pTAL-SEAP DNA (Clontech, Palo Alto, CA) using the LipofectAMINE Plus reagent. After 6 h of transfection, medium was replaced with fresh medium and cells were treated with RWE (50 µg/mL) for 48 h. The cell culture medium was harvested, centrifuged and supernatant was analyzed for SEAP activity, essentially as described by the manufacturer (Clontech, Palo Alto, CA), using a 96-well chemiluminescence plate reader.

### RNA Interference Ablation of AR in SAEC

Approximately 2×10^5^ SAEC were plated in 6-well plates and grown until ∼80% confluency. The cells were transfected with AR-siRNA (AAC GCA TTG CTG AGA ACT TTA) or scrambled siRNA (AAC ACG GCT TGA ATG ACT ATA; control) to a final concentration of 100 nM using RNAiFect™ transfection reagent (Qiagen). The cells were further incubated for 48 h at 37°C, and AR expression was determined by measuring AR protein by Western blot analysis using rabbit anti-AR polyclonal antibodies.

### Determination of inflammatory markers IL-6, IL-8 and PGE_2_

The SAEC were plated in 6-well plates at a density of 2×10^5^ cells per well in triplicate for each group. After 24 h, the cells were pretreated in serum-free SABM with or without zopolrestat (20 µM) or transfected with AR siRNA as described above. The growth-arrested and AR ablated cells were stimulated with RWE (150 µg/mL) for another 24 h in serum-free medium. The medium was collected from each well, centrifuged and supernatant was analyzed by using specific ELISA kits for IL-6 (Diaclone, Stamford, CT), IL-8 (R&D systems Inc, Minneapolis, MN) and PGE_2_ (Cayman Chemical Co., Ann Arbor, Michigan) according to the manufacturer's instructions.

### Determination of gene expression of IL-6, IL-8 and COX-2 by RT-PCR

The SAEC were grown in 6-well plates at a density of approximately 2×10^5^ cells per well. After approximately 80% confluence, cells were serum-starved in the presence or absence of zopolrestat (20 µM) for 24 h and then stimulated with 150 µg/ml RWE for 6 h. Total RNA from SAEC was isolated by using RNeasy kit (Qiagen) as per supplier's instructions. Aliquots of RNA (0.5–1.0 µg) isolated from each sample were reverse transcribed with Omniscript and Sensiscript reverse transcriptase one-step RT-PCR system with HotStar Taq DNApolymerase (Qiagen) at 55°C for 30 min followed by PCR amplification. The oligonucleotide primer sequences were as follows: 5′-ATGAACTCCTTCTCCACAAGCGC-3′ (sense) and 5′-GAAGAGCCCTCAGGCTGGACTG-3′ (antisense) for IL-6; 5′-ATGACTTCCAAGCTGGCCGTGGCT-3′ (sense) and 5′-TCT CAGCCCTCTTCAAAAACTTCTC-3′ (antisense) for IL-8; 5′-TGAAACCCACTCCA AACACAG-3′ (sense) and 5′-TCATCAGGCACAGGAGGAAG-3′ (antisense) for COX-2; and 5′-ATCTGGCACCACACCTTCTACAATGAGCTGCG-3′ (sense) and 5′-CGTC ATACTCCTGCTTGCTGATCCACATCTGC- 3′ (antisense) for β-actin. PCR was carried out in a PCR Sprint thermal cycler (Thermo electron corporation, Milford, MA) under the following conditions: initial denaturation at 95°C for 15 min followed by 35 cycles of 94°C for 1 min, 62°C for 1 min, 72°C for 1 min, followed by 72°C for 10 min for final extension. PCR products were electrophoresed with 1.5% agarose-1X TAE gels containing 0.5 µg/ml ethidium bromide. The densitometric analyses of the blots were performed by using Kodak 1D image analysis software.

### Western blot analysis

Forty micrograms of cytoplasmic protein extracts, prepared as described earlier were resolved on 10% SDS-PAGE. After electrophoresis, the proteins were electro transferred to a nitrocellulose membrane, blocked with 5% nonfat milk in TBST, and probed with antibodies against COX-2, iNOS, Bcl-XL, Bax, cyclin D1, E2F2 (1∶1,000 dilution) and GAPDH (1∶10,000 dilution) for 2 h. The blots were then washed, exposed to HRP-conjugated secondary antibodies (1∶5,000 dilution) for 1 h, and the antigen-antibody complex was detected by enhanced chemiluminescence (Amersham Pharmacia Biotech, Piscataway, NJ, USA).

### Animals

BALB/c mice were purchased from Harlan Sprague-Dawley (San Diego, CA, USA). All animal experiments were performed according to the National Institutes of Health Guide for Care and Use of Experimental Animals and approved by University of Texas Medical Branch Animal Care and Use Committee.

### Sensitization and challenge of animals

Eight-weeks-old female animals were sensitized with RWE as we have previously described [21], [25], [28]. Briefly, mice were sensitized with two intraperitoneal administrations of endotoxin-free RWE 150 µg/100 µl, combined with Alum adjuvant in a 3∶1 ratio, on days 0 and 4. On days 9 and 10, animals were treated with AR inhibitor (i.p. 25 mg/kg body weight) every 12 h for total duration of 48 h. On day 11, parallel groups of mice (*n* = 6–8) were challenged intranasally with RWE (100 µg), along with AR inhibitor. Control groups of mice were challenged with equivalent volumes of PBS.

### Evaluation of allergic inflammation

To evaluate inflammation, animals from all experimental groups were euthanized on day 14 with ketamine (135 mg/kg body wt) and xylazine (15 mg/kg body wt), and the lungs were lavaged with two 0.8 ml aliquots of ice-cold PBS. The cells were collected by centrifugation (1000 *g*, for 10 min at 4°C), re-suspended in one ml PBS and total cell counts were determined.

Differential cell counts were performed on cytocentrifuge preparations stained with hematoxylin and eosin. After bronchoalveolar lavage (BAL), the lungs were fixed with 4% paraformaldehyde, embedded in paraffin, and sectioned to 5 µm. Lung sections were stained with hematoxylin and eosin [25]. Perivascular and peribronchial inflammation and cell composition in the BAL were evaluated by a pathologist, blinded to treatment groups, to obtain data for each lung. The representative fields were photographed with a Photometrix CoolSNAP Fx camera mounted on a NIKON Eclipse TE 200 UV microscope.

Mucin production by the epithelial cells was assessed by periodic acid Schiff (PAS)-staining of formalin-fixed, paraffin-embedded lung sections. The stained sections were analyzed as above and representative fields were photographed with a Photometrix CoolSNAP Fx camera mounted on a NIKON Eclipse TE 200 UV microscope [25], [28].

To determine mucin levels, BAL was centrifuged at 12,000 rpm for 10 min at 4°C, and the supernatants were kept at −80°C until assayed. MUC5ac levels in the BAL were assessed by ELISA using commercially available anti-MUC5ac monoclonal antibody (I-13M1) (Lab Vision, Fremont, CA, USA). Briefly, MUC5ac present in the BAL was captured to a microtiter plate and a second antibody conjugated to biotin was added. After 30 min incubation with streptavidin-horseradish peroxidase (HRP) plates were washed and peroxidase substrate was added to obtain colorimetric product, which was quantified by spectrometry. Data are expressed as arbitrary units relative to a MUC5ac standard curve that was included on each plate [25].

Airway responsiveness was measured in unrestrained, conscious mice 3 days after the last challenge. Mice were placed in a barometric plethysmographic chamber, and baseline readings were taken and averaged for 3 min. Aerosolized methacholine in increasing concentrations (from 10 to 80 mg/ml) were nebulized through an inlet of the main chamber for 3 min. Readings were taken and averaged for 3 min after each nebulization and ‘enhanced pause’ (PENH) was determined. PENH, a dimensionless value, represents a function of the ratio of peak expiratory flow to peak inspiratory flow and a function of the timing of expiration, was calculated as (expiratory time/relaxation time^−1^)×(peak expiratory flow/peak inspiratory flow) according to the manufacturers' protocol. PENH correlates with pulmonary airflow resistance or obstruction and was used as a measure of airway responsiveness to methacholine.

### RNA isolation

Total RNA was isolated from homogenized lungs of sensitized, RWE-challenged mice (with and without ARI) using Ambion's RNAqueous Kit (Ambion, Austin, TX) according to manufacturer' commendations. RNA concentration was quantified using a DU530 Beckman spectrophotometer (Beckman Coulter, Fullerton,CA) at 260 nm. The 260/280 nm absorbance ratio was used to assess the purity of isolated RNA.

### Quantitative RT-PCR

One microgram of total RNA from each sample was transcribed into first-strand cDNA using SYBR GreenER two step qRT-PCR kit from ABI Prism (Invitrogen, Carsbad, CA). For selected genes forward and reverse primers were purchased from SABiosciences Corporation (Frederick, MD). Interleukin 3 (Cat # PPM03012E); Interleukin 4 (Cat # PPM03013E); Interleukin 5 (Cat # PPM03014E); Interleukin 8 receptor (Cat # PPM05308E0), Interleukin 10 (Cat # PPM03017B); Interleukin 13 (Cat # PPM03021A); Interleukin 17B (Cat # PPM03540A); Interleukin 18 (Cat # PPM03112B ); Chemokine (C-C motif) ligand 4 (Cat # PPM02948F); Chemokine (C-C motif) ligand 5 (Cat # PPM02960E ); tumor necrosis factor alpha (Cat # PPM03113F); Interferon gamma (Cat# PPM03113F). Internal controls were hypoxanthine guanine phosphoribosyl transferase 1 (Cat # PPM03559E) and glyceraldehyde-3-phosphate dehydrogenase (Cat # PPM02946E). PCR was conducted under the following conditions: denaturation at 95°C for 10 min, 1 cycle, followed by 40 cycles of denaturation at 95°C for 15 s, annealing and elongation at 60°C for 60 s. The levels of RNA for the target sequences were determined by melting curve analysis using the ABI PRISM 7000 sequence Detector software (Applied Biosystems, Foster City, CA).

### Statistical analysis

For the cell culture experiments data presented are mean±SD and P values were determined by unpaired, two-tailed Student's t test. For animal studies, data collected from *in vitro* and *in vivo* experiments were analyzed by ANOVA, followed by Bonferroni post-hoc analyses for least significant difference. For quantitative RT-PCR data statistical analysis was done by unpaired, two-tailed t-test using Graph-pad prism software. *p*<0.05 was considered as statistically significant.
